# Anticancer Effects with Molecular Docking Confirmation of Newly Synthesized Isatin Sulfonamide Molecular Hybrid Derivatives against Hepatic Cancer Cell Lines

**DOI:** 10.3390/biomedicines10030722

**Published:** 2022-03-20

**Authors:** Mahmoud Eldeeb, Eman F. Sanad, Ahmed Ragab, Yousry A. Ammar, Khaled Mahmoud, Mamdouh M. Ali, Nadia M. Hamdy

**Affiliations:** 1 Department of Biochemistry, Biotechnology Research Institute, National Research Centre, 12622 Giza, Egypt; me.eldeeb@nrc.sci.eg (M.E.); dr-mamdouh-moawad@nrc.sci.eg (M.M.A.); 2 Biochemistry and Molecular Biology Department, Faculty of Pharmacy, Ain Shams University, 11566 Cairo, Egypt; dr.emansanad@pharma.asu.edu.eg; 3 Department of Chemistry, Faculty of Science (for Boys, Cairo Branch), Al-Azhar University, 11884 Cairo, Egypt; ahmed_ragab@azhar.edu.eg (A.R.); yossry@azhar.edu.eg (Y.A.A.); 4 Department of Pharmacognosy, Pharmaceutical and Drug Industries Research Institute, National Research Centre, 12622 Giza, Egypt; km.hanafi@nrc.sci.eg

**Keywords:** apoptosis, HepG2, Huh7, isatin sulfonamides, angiogenesis, invasion, cancer hallmarks, molecular docking, EGFR tyrosine kinase inhibitor

## Abstract

The current study investigated the cytotoxic effect of ten sulfonamide-derived isatins, following molecular hybridization, based on the association principles, on hepatocellular carcinoma (HCC) HepG2 and Huh7 cell lines, compared for safety using human normal retina pigmented epithelial (RPE-1) cells. The ten compounds showed variable in vitro cytotoxicity on HepG2 and Huh7 cells, using the MTT assay. Four compounds (4/10) were highly cytotoxic to both HepG2 and HuH7. However, only 3 of these 4 were of the highest safety margin on RPE-1 cells in vitro and in the *in vivo* acute (14-day) oral toxicity study. These later, superior three compounds’ structures are 3-hydroxy-3-(2-oxo-2-(p-tolyl)ethyl)-5-(piperidin-1-ylsulfonyl)indolin-2-one (**3a**), N-(4-(2-(2-oxo-5-(piperidin-1-ylsulfonyl)indolin-3-ylidene)acetyl)phenyl)acetamide (**4b**), and N-(3-(2-(2-oxo-5-(piperidin-1-ylsulfonyl)indolin-3-ylidene)acetyl)phenyl)acetamide (**4c**). The half-maximal inhibitory concentration (IC50) of the tested compounds (**3a**, **4b**, and **4c**) on HepG2 cells were approximately 16.8, 44.7, and 39.7 μM, respectively. The **3a**, **4b**, and **4c** compounds significantly decreased the angiogenic marker epithelial growth factor receptor (EGFR) level and that was further confirmed via molecular docking inside the EFGR active site (PDB: 1M17). The binding free energies ranged between −19.21 and −21.74 Kcal/mol compared to Erlotinib (−25.65 Kcal/mol). The most promising compounds, **3a**, **4b**, and **4c**, showed variable anticancer potential on “hallmarks of cancer”, significant cytotoxicity, and apoptotic anti-angiogenic and anti-invasive effects, manifested as suppression of Bcl-2, urokinase plasminogen activation, and heparanase expression in HepG2-treated cells’ lysate, compared to non-treated HepG2 cells. In conclusion, compound “**3a**” is highly comparable to doxorubicin regarding cell cycle arrest at G2/M, the pre-G0 phases and early and late apoptosis induction and is comparable to Erlotinib regarding binding to EGFR active site. Therefore, the current study could suggest that compound “**3a**” is, hopefully, the most safe and active synthesized isatin sulfonamide derivative for HCC management.

## 1. Introduction

“Hallmarks of cancer” include the programmed molecular cell death mechanism “Apoptosis” [1]. Apoptosis is related to other cancer hallmarks such as progression and metastasis [1,2,3]. Therefore, it is important to design more drug moieties targeting apoptosis and/or tumor invasiveness.

Isatin (1H-indole-2,3-dione) is a well-known natural product found in many plants which has also been a common scaffold in various anticancer drugs [4,5]. Many isatin derivatives display diverse pharmacological activities, including anti-viral, anti-convulsant, anti-bacterial, and anti-fungal activities [6,7,8]. Because of its unique size and privileged electronic properties, there is considerable interest in pharmaceutical drug development and the biochemical pharmacology of isatin anticancer drug derivatives [9]. The isatin scaffold is incorporated in many synthetic anti-cancer drugs such as Sunitinib V maleate and Toceranib phosphate [10]. These inhibitors with isatin moiety have shown outstanding anti-cancer effects in clinical trials, including multi-receptor tyrosine kinase inhibitor activity [11]. Therefore, anti-tumor drugs containing isatin scaffolds could possess a broader spectrum of cytotoxicity against cancer cells, namely, apoptotic, anti-proliferative, and anti-migratory effects, collectively, anti-tumor invasiveness.

The α, β-unsaturated ketone (Michael Acceptor) pharmacophore is presented as a common skeleton in many naturally occurring agents including isatin [10,12]. Therefore, incorporating a “Michael Acceptor” into designed molecules results in products with improved cytotoxicity [10,13]. Hence, “α, β-unsaturated ketone” can be considered as a cytotoxic drug design “a functionality structure”.

Molecular hybrids are one of the most popular strategies to develop new anticancer agents, based on combining structural features of two different active fragments. This reduces the risk of drug–drug interactions and improves the biochemical/molecular outcome(s) [14,15]. During the last decade, many isatin-based hybrids have been developed as promising anti-cancer agents, including Erlotinib, Vandetanib, Olaparib, and others [16]. Several published datasets have reported that isatin derivatives have potential toward different biological targets such as tubulin, tyrosine kinase, and histone deacetylase, leading to apoptosis and, moreover, influencing apoptosis-related gene expression [17,18,19,20]. Interestingly, incorporation of sulfonamide moiety to a benzene ring of isatin showed increased anti-tumor activities with a potential inhibitory effect against epidermal growth factor receptors (EGFRs) [21,22].

This study aimed to characterize ten novel synthesized isatin sulfonamide-molecular-hybrid derivatives to target EGFRs and evaluate their in vitro antiproliferative activities. Based on the previous promising anti-tumor activities of some morpholinosulfonyl isatin derivatives on HepG2 [22,23], we tested their anti-cancer activities against two hepatocellular carcinoma (HCC) cell lines: the HepG2 and Huh7 cell lines, which are different in their drug-metabolizing activities as well as their p53 expression. To ensure the safety of the tested compounds, the most active derivatives were tested in vitro against normal human cell line, and an in vivo acute oral toxicity study was performed. The most active and safest synthesized compounds have been evaluated for their possible apoptotic effect, cellular cytotoxicity, and whether they cause angiogenesis arrest or invasion inhibition. The cell death mechanism underlying this activity was investigated via cell cycle analysis and apoptotic studies. Doxorubicin (Dox) was used as an internationally accepted cytotoxic agent to be the positive control to compare the cytotoxic potential of the tested drugs. In addition, inhibitory mechanism confirmation using molecular docking (MD) was carried out to study the interaction of promising compounds with EGFRs.

## 2. Materials and Methods

### 2.1. Biochemical Reagents, Chemicals, Solvents

Unless otherwise specified, chemicals and reagents were purchased from Sigma-Aldrich Chemical Co. (St. Louis, MO, USA) and were of analytical grade. Dimethyl sulfoxide (DMSO) was used as the vehicle for isatin sulfonamide derivatives per their molecular wt. Dox was used as a small anti-cancer cytotoxic reference drug (positive control), Erlotinib as reference EGFR inhibitor. Other chemicals and solvents such as xylene, paraffin, trypsin, and alcohol were of the highest grade commercially available.

### 2.2. Biochemical/Molecular Assay Kits

MTT assay kit and propidium iodide (PI) staining FC assay kit was purchased from Abcam (Boston, MA, USA). AST, ALT, and bilirubin (total and direct) kits and creatinine, urea kits, and GSH kit were all purchased from Biodiagnostics Company (Cairo, Egypt).

Heparanase ELISA kit (Abcam, Cambridge, UK), Annexin V/PI double staining kit (eBiosciencesDx), EGFR 96-well plate kit (Abcam, Cambridge, UK), uPA ELISA kit (Creative Diagnostics, New York, NY, USA), and BcL-2 ELISA kit (Zymed Laboratories, Carlsbad, CA, USA) were used.

### 2.3. Cell Lines

The cell lines used were obtained from ATCC including Hep G2 [HEPG2] (ATCC HB-8065), Huh7 cell line-615, and the normal retina pigmented epithelial cells immortalized with hTERT; hTERT RPE-1 (ATCC CRL-4000). Handling procedure for flask cultures and subculturing, media (DMEM, bovine serum albumin), and incubations were all performed according to www.ATCC.org (accessed on April 2021).

### 2.4. Animals

5–7-week-old mice (weighing 15–20 g, females) were obtained from Nile Co. for Pharmaceutical and Chemical Industries (Egypt). Mice were acclimatized for 1 week under standard laboratory conditions in cages in a room with 12 h light/dark cycles, at 25 °C  ±  2 °C and 55%  ±  5% relative humidity. Mice were fed on standard diet pellets (El Nasr Company for Intermediate Chemicals, Giza, Egypt) containing no less than 20% protein, 3.5% fat, 6.5% ash, 5% fiber, and a vitamin mixture. Animals were allowed free access to drinking water ad libitum.

### 2.5. Isatin Sulfonamide Derivative Synthesis

In this study, motivated by the structural features of isatin and α, β-unsaturated ketone, we proposed combining these two bioactive scaffolds into a single chemical entity. We centered on molecular hybridization strategy [23] to design and synthesize a novel series of isatin sulfonamide derivatives carrying the α, β-unsaturated ketone scaffold.

Quantitative Structure–Activity Relationships (QSAR) model was utilized to get a more profound understanding of the molecular description of compounds’ activities. Few QSAR models that clarify the anti-cancer activity of isatin analogues were reported [24].

### 2.6. Chemical Synthetic Pathway

**Figure 1:**
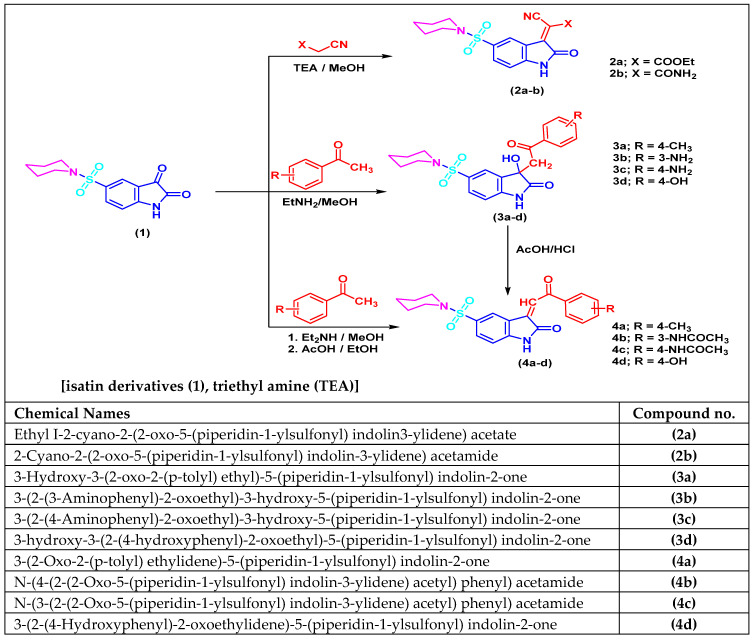
Synthetic routes scheme for arylidine and 3-hydroxy-3-substituted isatin sulfonamide and 2-oxindole as well as 3-phenacylidene-2-indolinone derivatives with their chemical names.

The routes adopted to synthesize isatin sulfonamide derivative compounds were as described in Figure 1. The parent molecule is 5-(piperidin-1-ylsulfonyl) indoline-2, 3-dione. This compound was derivatized into three compounds: **2a**-**b**, **3a**-**d**, and **4a**-**d**. These newly designed compounds were structurally recognized and affirmed by using different spectroscopic analysis as IR, ^1^H NMR, and ^13^C NMR spectra as described in organic synthesis literature article [25]. Additionally, all the spectra investigations (in the Supplementary File) and were in great conformity with the proposed structures. These compounds were prepared by the Organic Chemistry authors in the Organic Chemistry Lab, Faculty of Science (Boys-Branch), Al Azhar University, Cairo, Egypt. Then, these three compounds were derivatized to produce ten final isatin sulfonamide-derivative compounds to be tested further by various biochemical/molecular assays.

### 2.7. Cancer Cell Proliferation Inhibition

MTT-based cell viability assay was carried out to evaluate the antiproliferation potential of the target compounds on HCC cell lines (HepG2 and Huh7). The half maximal inhibitory concentration (IC50) values were calculated against DMSO non-treated HepG2 cells (negative control) and Dox-treated HepG2 cells (positive control). The assay was conducted using 3-(4, 5-di-MethylThiazol-2-yl)-2,5-diphenyl Tetrazolium bromide (MTT) to test cell viability, according to the method described by Mosmann [26] with minor changes [27]. The yellow MTT is reduced in the viable cells to purple formazan. The insoluble formazan is solubilized by DMSO to colored solution measured spectrophotometrically. Briefly, cells were seeded in a 96-well plate at density of 1 × 10^4^ cells/well for 24 h in 5% CO_2_ incubator at 37 °C to allow their adherence. Then, cells were incubated for 48 h with different concentrations of test compounds ranging from 100 to 1.56 µM in 5% CO_2_ incubator at 37 °C. After incubation, 20 μL of a 5 mg/mL MTT solution were mixed with the contents of each well, and the plate was incubated for another 4 h. Then, the medium was suctioned, and the wells were washed with PBS. After 2 h of drying, 200 μL of DMSO was added to each well. The plate was set on a shaker to dissolve the formazan crystals. Then, the absorbance was measured spectrophotometrically at 570 nm with a reference wavelength of 630 nm using ELX800 UV universal microplate reader (BioTek Instruments Inc., VT, Santa Clara, CA, USA).

All experiment conditions were tested in three replicates. The % cell viability was calculated from the following equation: (mean absorbance of treated sample/mean absorbance of negative control sample) × 100. The % death rate = 100 -% cell viability, and the inhibitory concentration of 50% (IC50) was measured from the exponential curve of viability against concentration (dose–response curve) [28].

### 2.8. In Vitro Safety/Cell Viability Assay towards Human Healthy RPE1 Cell Line

Toxicity of the most active six isatin sulfonamide derivatives (**2a, 3a, 4a, 4b, 4c, 4d**) were evaluated using noncancerous cells (RPE1) to examine the safety of these newly synthesized isatin sulfonamide derivatives towards normal human cell line, again using the MTT test.

### 2.9. In Vivo Acute Oral Toxicity Study

GPower 3.0.10 software was used to estimate the sample size needed for the experiment and the number of mice per group but, according to the Organization for Economic Cooperation and Development (OECD) Test Guidelines No. 407 (OECD, 2008 and updated 2017) [29] and under the International Academy of Science’s Guide for the Care and Use of Laboratory Animals, as well as the ARRIVE guidelines, to use the least number of mice per group for the acute single dose oral toxicity test.

### 2.10. Ethics Statement

Animal care and all experimental protocols were approved and conducted in accordance with the ethical guidelines approved by the Institutional Review Board of the Faculty of Pharmacy, Ain Shams University, Cairo, Egypt (Ethics Committee Approval, 10/2019/1).

### 2.11. Experimental Design

Animals were divided randomly into nine groups (5 animals per group). Before the experiment, the body weight (BW) of all mice was recorded. All mice were made to fast for 24 h and treated once (day zero) then carefully monitored for 14 days.

Normal control group: mice received saline orally once;

Negative control group: mice received vehicle (DMSO) orally once;

Positive control group: mice were injected with Dox (15 mg/kg BW, i.p.) once [30,31].

Isatin-sulfonamide-derivative-compound-treated groups (**2a**, **3a**, **3d** and **4b**, **4c** and **4d**) of mice received 250 mg/kg BW of each orally once [29,32].

After single oral dose, animals were observed for the first 30 min and 4–5 times at intervals of 48 h to record any signs of abnormality. The animals’ BWs were recorded at the end of the 14 days of observation. Blood samples were collected from the retro-orbital plexus and allowed to clot. Sera were prepared by centrifugation at 4000 rpm for 15 min and then kept frozen at − 80 °C for liver and kidney function tests (LFTs and KFTs). Thereafter, mice were deprived of food overnight, euthanized, and sacrificed by cervical dislocation. Liver and kidney tissues were collected, washed with ice-cold saline, and weighed. Excised weighed livers and kidneys were fixed in a suitable buffer for histological examination.

N.B. the objective of the acute toxicity study was not the determination of LD50 values.

### 2.12. Histopathological Examination

Liver and kidney tissues were fixed in 10% neutral buffered formaldehyde overnight and then embedded in paraffin, deparaffinized in serial grades of alcohols, cleared in xylene, and were subjected to ultramicrotomy, where 4 μm thick tissue sections were cut by rotatory microtome. Tissue sections were stained with hematoxylin and eosin (H and E) for histological examination using full HD light microscopic imaging system (Leica Microsystems GmbH, Wetzlar, Germany). Standard procedures for sample fixation and staining were done as previously described [33,34].

### 2.13. Flow Cytometric Analysis and Apoptotic Studies

Targeting cell cycle and induction of apoptosis are promising strategies to develop potential anticancer agents. Therefore, the 3 promising isatin sulfonamide derivatives were investigated for their apoptotic activities.

#### 2.13.1. Cell Cycle Analysis

In 6-well plate, HepG2 (1 × 10^5^ cells per well) were treated with either (0.1% DMSO) vehicle or 5 μM of **3a**, **4b**, and **4c** compounds. After 48 h incubation, cells were harvested and fixed for 12 h using ice-cold 70% ethanol at 4 °C. Ethanol was removed and cells were washed by PBS and incubated for 30 min at 37 °C in 0.5 mL of PBS containing 1 mg/mL RNase. Then, cells were stained for 30 min with PI in the dark and the DNA content was determined using flow cytometer [35].

#### 2.13.2. Annexin V/FITC Apoptosis Assay

To apprehend the mechanism of the chosen newly synthesized isatin sulfonamide-derivative compounds and to recommend further in vivo testing as new chemotherapeutics, HepG2 cells were incubated with IC50 dose of compounds **3a**, **3b**, and **3c**, against the positive chemotherapeutic control, Dox. Annexin V/PI double staining kit (eBiosciencesDx) was used to detect apoptotic activity of the selected isatin sulfonamide derivatives. The Annexin V corresponding signal provides a very sensitive marker for cellular apoptosis, while PI was used to detect necrotic or late apoptotic cells, identified by the loss of plasma integrity and loss of nuclear membranes.

Briefly, 1 × 10^5^ cells/mL of HepG2 cells were seeded into a 6-well plate and incubated at 37 °C, 5% CO_2_, overnight. Cells were treated with either 0.1% DMSO or IC50 of the three selected compounds for 48 h (50 μL from each compound solution in DMSO; IC50 dose) in triplicate. After time elapsed, trypsinization of cells was carried out. Washing was done by PBS once, followed by fixation using 70% ethanol. Fixed cells were stored at 4 °C for 2 h, then centrifuged at 500× *g* for 5 min. Again, cells were washed with PBS prior to staining. An amount of 200 μL of the staining solution (annexin V- fluorescein 5-isothiocyanate (FITC) and PI in binding buffer (10 mM HEPES, 140 mM NaCl, and 2.5 mM CaCl_2_ at pH 7.4)) were added to the cell suspension and incubated at 37 °C for 20 to 30 min in dark and analyzed with a fluorescence-activated cell sorter (FACS) (BD FACSCalibur, BD Biosciences, California, USA). Data were analyzed using ModFit LT v2.0 (Verity Software House, Topsham, Maine, USA).

### 2.14. Biochemical Evaluation

As previously described [36], with little modification, HepG2 cells were cultured in T25 flask until they reached 30% confluency. Then, cells were incubated separately with selected isatin derivatives (**3a**, **4b**, and **4c**) at their IC50 for 48 h at 37 °C under 5% CO_2_. After treatment, cells were washed by PBS, trypsinized and centrifuged at 10,000 rpm. The formed pellets were washed twice with PBS and lysed in 1 mL ice-cold radio immunoprecipitation assay (RIPA) lysis buffer (50 mM Tris-HCl, pH 8.0, 150 mM NaCl, 1% Triton X-100, 0.5% sodium deoxycholate, 0.1% sodium dodecyl sulfate) and kept on ice for 20 min. Then, the mixture was centrifuged at 15,000 rpm for 15 min to remove any cell debris. The cell lysate was aliquoted and stored at −80 °C for determination of protein using Pierce BCA Protein Assay Kit (Thermo Fisher Scientific, Waltham/Boston, USA) according to manufacturer’s recommendations and determination of other biochemical parameters including EGFR, urokinase Plasminogen Activator (uPA), B-Cell Lymphoma-2 (Bcl-2), heparanase, and oxidative stress markers (glutathione; GSH and malonaldehyde; MDA).

Levels of EGFR [37] and heparanase [38] in cell lysate were investigated using commercially available enzyme-linked immunosorbent assay (ELISA) kits supplied by Abcam (Cat. No. ab269558, ab256401, respectively). Levels of uPA [39] were measured using ELISA kit supplied by Creative Diagnostics (New York, USA, Cat. No. DEIA1630). Bcl-2 levels [36,40] were determined using ELISA kit supplied by Zymed Laboratories, Carlsbad, California (Cat. No. 99-0042). Briefly, standards and samples were added to corresponding 96-well plate coated with suitable antibody. After the washing step, biotinylated anti-human antibody was added to each well. After washing away unbound biotinylated antibody, horseradish peroxidase (HRP)-conjugated streptavidin was pipetted into each well. A peroxidase substrate solution was added to all wells, after washing, and the color developed was proportionate to the amount of EGFR bound. Finally, the stop solution was added and the color intensity was measured at 450 nm.

Glutathione levels were determined using GSH Biodiagnostics kit (Cairo, Egypt). The method is based on the reduction of 5,5′dithiobis (2-nitrobenzoic acid) (DTNB) with glutathione (GSH) to produce a yellow compound. The reduced chromogen is directly proportional to GSH concentration and its absorbance was measured at 405 nm [41,42].

Levels of MDA, the marker of lipid peroxidation, were measured by thiobarbituric acid (TBA) method. TBA reacts with MDA in an acidic medium at temperature of 95 °C for 30 min to form colored thiobarbituric acid reactive (TBAR) product, the absorbance of which was measured at 534 nm. The MDA kit was purchased from Biodiagnostics, Cairo, Egypt [43,44].

### 2.15. Molecular Docking Simulation

MD was performed using Molecular Operating Environmental (MOE) software (Version10.2008, Chemical Computing Inc., Montreal, Quebec, Canada) targeting EGFRs. The macromolecule structure for the EGFR tyrosine kinase domain inhibitor Erlotinib (PDB: 1M17) was obtained from the protein data bank, February 2021 (https://www.rcsb.org/structure/1M17) (accessed on 5 February 2022). The docking process and the generation of the active site were performed according to the previously reported method [22,40,45]. The structure of the newly designed and most promising compound was drawn using ChemDraw14.0 then exported to MOE. The new structure was potentate 3D and minimized energy using MMFF94x force field. The co-crystalized ligand was exposed to validation process with RSMD 1.99 Å using Triangle Matcher placement method and London dG as docking score energy. Validation process of the co-crystalized ligand Erlotinib showed binding energy S = −25.65 Kcal/mol with RMSD = 0.89 °A Å, through one hydrogen bond backbone donor, between Met769 and nitrogen of quinazoline, with bond length 3.04 Å and strength 27%.

### 2.16. Statistical Analysis

Data are presented as the mean ± SEM. All experiments were performed in triplicate and repeated twice. Testing of data normality was carried out using Kolmogorov–Smirnov test. Multiple comparisons were done using a one-way ANOVA test followed by Tukey–Kramer as a post hoc test. The statistical significance criterion used was the 0.05 level of probability *p*. Statistical analyses were carried out using GraphPad Prism v 5 software (ISI software, San Diego, California, USA).

## 3. Results

### 3.1. Biological Evaluation

#### 3.1.1. Antiproliferative and Anticancer Activities of Isatin Sulfonamide Derivatives on HepG2 and Huh7 Cell Lines

The cytotoxic effect of the ten compounds was assessed by the MTT assay using two human hepatocellular carcinoma cell lines, HepG2 and Huh7. The assay was performed in triplicate to calculate the IC50 (µM), the concentration required for 50% cell cytotoxicity and a % death rate of 100 p.p.m (µg/mL) after the specified time (48 h) against DMSO (vehicle, negative control) and Dox (positive control drug) (Table 1). The compounds showed more selectivity to HepG2. Tumor cells showed normal growth in the culture system, and DMSO did not seem to have any noticeable effect on cellular growth. Only six compounds (**2a**, **3a**, **4a**, **4b**, **4c**, and **4d**) had cytotoxic activity compared to the DMSO-treated cells (HepG2 or Huh7).

Before proceeding further to test these latter six compounds as potential selective anticancer agents, their safety was examined in vitro and in vivo.

#### 3.1.2. In Vitro Safety Assay towards Human Healthy Retina Pigmented Epithelial (RPE1) Cell Line

As shown in Table 1, three superior isatin sulfonamide derivatives (**3a**, **4b**, and **4c**) showed a non-cytotoxic effect towards the noncancerous cell line (RPE1), proving a high safety profile as ***potential selective anticancer agents*** to be tested later. In addition, compound **4c** was better than **4b** and **3a** regarding both the safety and death rate on HepG2. However, **4b** and **4c** showed lower IC50 and higher death rates than **3a** on Huh7 cells (Table 1).

The results were expressed as MTT assay IC50 (µM) and % cell viability at 100 ppm values (Table 1). The three superior isatin sulfonamide derivatives (**3a**, **4b**, and **4c**) showed non-cytotoxic effects towards the noncancerous cell line (RPE1), thereby proving a high safety profile as potential selective anti-cancer agents to be tested further.

#### 3.1.3. In Vivo Acute Oral Toxicity Assay

**Figure 2:**
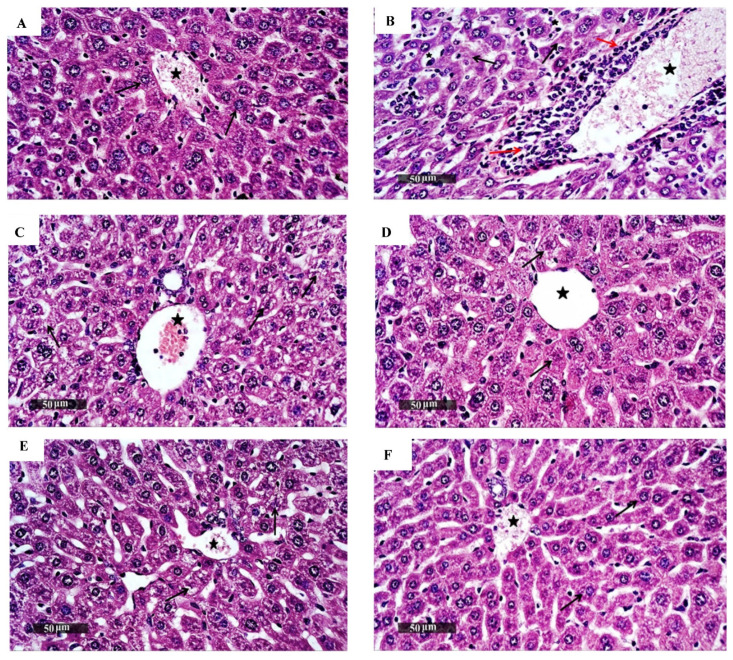
Photomicrographs of mice liver sections stained by H and E from (**A**) the normal control group, which demonstrated normal histological features of the liver parenchyma with apparently intact well-organized hepatocytes with intact subcellular details (black arrow), normal hepatic sinusoids, vasculatures (star), and portal tracts; (**B**) Dox-treated mice as the positive control group, which showed hepatocellular necrotic changes (arrow) accompanied with moderate dilatation of the hepatic blood vessels and hepatic sinusoids (star) associated with focal perivascular mixed inflammatory cell infiltrates (red arrow); (**C**) mice treated with compound 3a, which showed intact vasculatures (star) as well as hepatic sinusoids with no inflammatory cell infiltrates; (**D**) mice group treated with compound 4b, which demonstrated apparently intact hepatocytes and allover hepatic parenchyma with intact vasculatures (star), and almost no record of inflammatory cell infiltrates; (**E**) mice treated with compound 4c, and (**F**) negative control group that received DMSO. Both (**E**,**F**) groups showed almost intact histological features of hepatic parenchyma (as normal control; (**A**)). [scale bars 50 µm].

**Figure 3:**
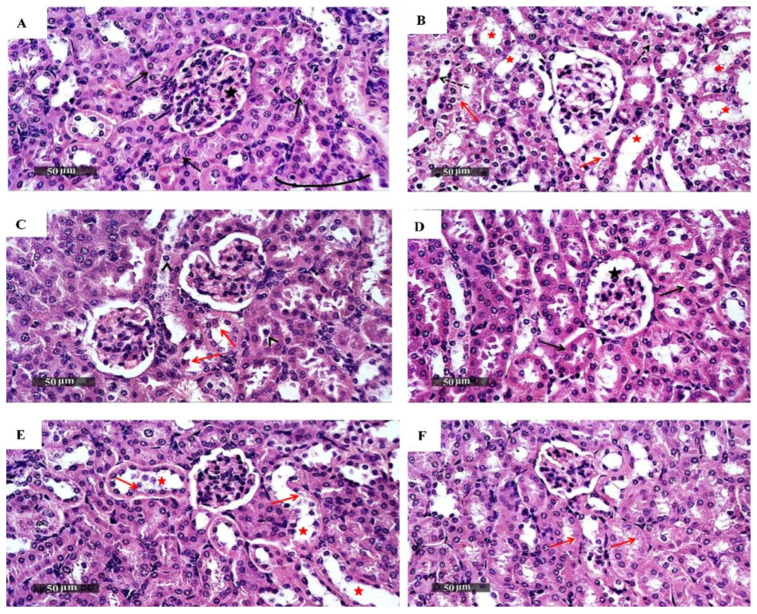
Photomicrographs of mice kidney sections, where renal tissue stained by H and E from (**A**), the normal control group, demonstrated normal histological features of the renal parenchyma with many intact renal corpuscles (star) and nephron segments with intact tubular epithelium (black arrow) and intact interstitial tissues and vasculatures. (**B**) Positive control treated with Dox, showing dilatation of the cortical tubular segments (red star) with tubular vacuolar degenerative changes (red arrow), loss of luminal border integrity, and occasional tubular epithelial necrotic cells records (dashed black arrow); (**C**) group treated with compound 3a, showing intact well-organized histological features of renal parenchyma with minimal records of sporadic tubular epithelial changes (red arrow); (**D**) group treated with compound 4b, with almost intact histological features of renal parenchyma; (**E**) group treated with compound 4c, where most of the tubular segments showed almost intact well-organized morphological features (as in (**A**)), and (**F**) negative control group that received DMSO, which showed almost intact histological features of renal parenchyma with minimal records of tubular degeneration (red arrow). [scale bars 50 µm].

After giving isatin sulfonamide-derivative compounds **2a**, **3a**, **4a**, and **4b**, **4d**, and **4c** as a single acute oral dose of 250 mg/kg [29,32], all the mice groups survived the treatment period (14 days). No physical or abnormal changes were observed in the body weight (BW), skin, fur, eyes, mucus membranes, tremors, salivation, behavior patterns, sleep patterns, or the animals’ overall appearance. Kidney and liver biochemical analyses (liver and kidney function tests) were reported as normal (Table 2). Histopathology analyses (H and E staining) of the liver and kidney tissues are presented in Figure 2 and Figure 3, respectively. There were no differences in the hepatic or renal tissue structures between the treated (**3a**, **4b**, and **4c** groups), the untreated normal mice control group, and the negative control group which received DMSO, and these were superior to the positive control Dox-treated animals, where hepatic and renal pathology were obvious.

#### 3.1.4. Isatin Sulfonamide Molecular Hybrids Effect Cell Cycle Analysis

Flow cytometry was done to characterize whether the synthetic isatin sulfonamide-derivative compounds exerted an anticancer effect via disrupting the cell cycle compared to untreated controls. Moreover, flow cytometry would prove whether isatin sulfonamide-derivative hybrids activate the programmed cell death pathway, inducing apoptosis, with an overall gain of “cell death”, or not. The results are presented in Table 3, where the % cell cycle arrest in the different cell cycle phases showed that treatment of HepG2 cells with compounds **3a** and **4c** arrested the cells at the G2-M phase by 44.58 and 37.62 %, respectively, which is 6.32- and 5.30-fold higher compared to the negative control, HepG2 (7.1%). It was also noticed that the effect of compound **3a** was superior to the effect of Dox (39%) on arresting the cells at the G2-M phase. In addition, compound **4b** caused the accumulation of cells at the G0-G1 and S phases by 41.25 and 53.26%, respectively. Interestingly, the three compounds, **3a**, **4b**, and **4c**, induced apoptosis at the pre-G1 phase, and the percentages of cell death were 46.29, 28.14, and 32.02, respectively, in comparison to the negative control (1.47%). Again, induction of apoptosis at the pre-G1 phase by compound **3a** was more than the induction induced by doxorubicin (46.29% versus 42.38%). These results indicate that compounds **3a**, **4b**, and **3c** could target the cancer cell cycle and induce apoptosis at different phases.

The cell cycle arrest in HepG2 is expressed as % cells in each phase after treatment with 5 µM isatin sulfonamide tested compounds, **3a**, **4b**, and **4c**, against positive and negative controls. The apoptotic activity of compounds **3a**, **4b**, and **4c** is expressed as % cell death induction after treatment of HepG2 with IC50 of the selected compounds for 24 h. The positive control was Dox-treated HepG2 cells; the negative control was DMSO-treated HepG2 cells.

#### 3.1.5. Effect of Isatin Sulfonamide Derivatives on Apoptotic Assay

**Figure 4:**
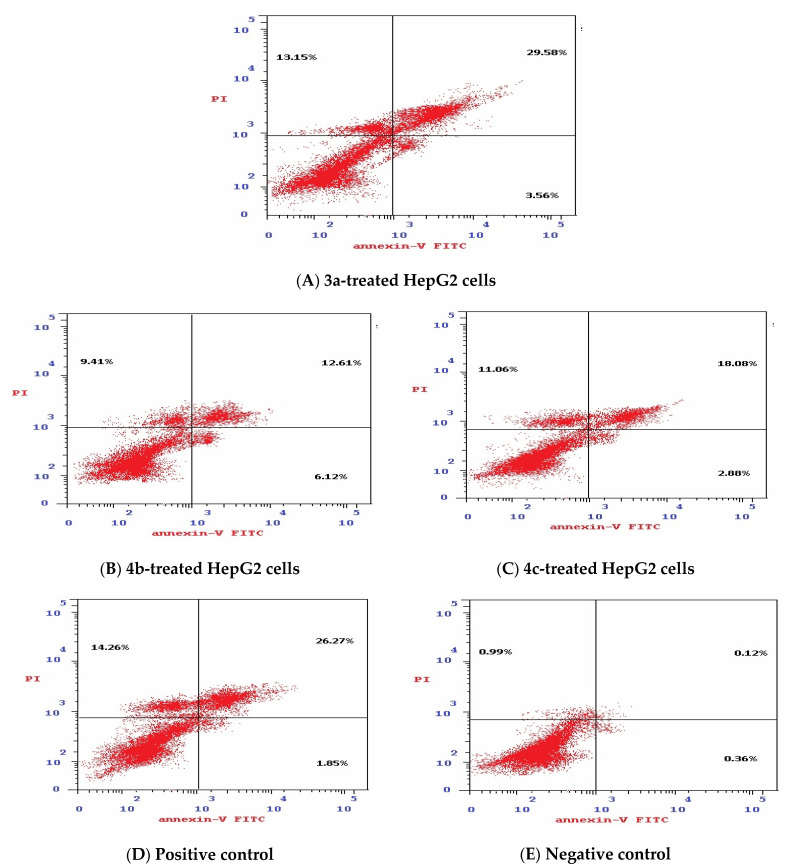
Apoptosis analysis by flow cytometry using the Annexin V/PI double staining method ((**A**) compound **3a**-treated HepG2 cells, (**B**) compound **4b**-treated HepG2 cells, (**C**) compound **4c**-treated HepG2 cells, (**D**) positive control; Dox-treated HepG2 cells, and (**E**) negative control; non-treated HepG2 cells). Apoptosis analysis depends on the quantitation of DNA content after PI staining, being stoichiometric, i.e., cells in the S phase will have more DNA than cells in the G1 phase and will take up proportionally more dye and will fluoresce more brightly. Cells in the G2 phase will be approximately twice as bright as cells in the G1 phase.

The mechanism of cell death was analyzed by flow cytometry using Annexin V/PI double staining. The data generated were plotted in two-dimensional dot plots, in which PI is represented versus Annexin V-FITC (Figure 4).

These plots were divided into four regions corresponding to:

(1) Viable cells, which are negative to both probes (PI/FITC −/−),
(2) Apoptotic cells, which are PI negative and Annexin positive (PI/FITC −/+),
(3) Late apoptotic cells, which are both PI and Annexin positive (PI/FITC +/+),
(4) Necrotic cells, which are PI positive and Annexin negative (PI/FITC+/−).

As shown in Table 4 and Figure 4, the % of total apoptosis induced by the tested compounds (**3a**, **4b**, and **4c**) were 46.3%, 28.1%, and 32.0%, respectively, compared to the negative control (1.47%). Compound 3a exhibited the highest induction of total apoptosis, 31.49-fold greater than that of negative control HepG2 cells and higher than the total apoptosis induced by Dox (42.38%). Moreover, compound **3a** was the most potent compound for inducing total apoptosis or necrosis (46.29 % and 13.15%, respectively) in comparison to **4b** and **4c**. In addition, the percentage of late apoptosis varies from 29.58% to 12.61% and 18.08% for compounds **3a**, **4b**, and **3c**, respectively, when compared to both negative control HepG2 cells (0.12%) and Dox-treated cells (26.27%).

#### 3.1.6. Effect of Isatin Sulfonamide Molecular Hybrids on Other Cancer Hallmark Markers Assay

As illustrated in Table 4, compounds **3a-**, **4b-**, and **4c-**treated HepG2 cells showed significant reductions in the EGFR levels, where **3a-**treated cells reported the most reduction to 42 ± 2.3 pmol/mg protein in comparison to untreated HepG2 cells (306 ± 20 pmol/mg protein). This signifies the potent anti-angiogenic effect of the selected promising isatin sulfonamide derivatives.

As clarified in Table 4, cells treated with compounds **3a**, **4b**, and **4c** showed significantly lower levels of uPA (1258 ± 15, 1916 ± 40, and 1729 ± 38 nmol/mg protein, respectively) than the non-treated negative control HepG2 cells (3149 ± 111 nmol/mg protein). These levels, except those for 3a-treated cells, were significantly higher than the Dox-treated cells uPA levels (1244 ± 18 nmol/mg protein).

Anti-apoptotic repression via Bcl-2 antigen level restoration was also evaluated. Compounds **3a, 4b, and 4c** showed significant differences in levels of Bcl-2 (4.2 ± 0.1, 2.5 ± 0.04, and 3.6 ± 0.11 nmol/mg protein, respectively) when compared to the negative control (7.8 ± 0.11 nmol/mg protein). This points to the fact that one role of these three selected isatin derived compounds as anti-cancer promising drugs is via apoptosis induction.

Heparanase expression levels were assessed as markers of metastasis. Compound 3a showed the lowest levels of heparanase (1262 ± 35 pmol/mg protein) in comparison to the negative HepG2 cell control (3097 ± 160 pmol/mg protein). Compounds **4b** and **4c** lowered heparanase expression levels in HepG2 cell lysates as well, to 1827 ± 30 and 1449 ± 12.5 pmol/mg protein, respectively.

With respect to markers of oxidative stress, the three investigated compounds had no significant effect on GSH levels compared to non-treated HepG2 cells. On the other hand, only compounds **3a** and **4b** had remarkable reducing effects on MDA levels in HepG2 cell lysates, to 2.0 ± 0.5 and 3.7 ± 0.4 µmol/mg protein, respectively, in comparison to both non-treated HepG2 cells (13.8 ± 1.0 µmol/mg protein) and Dox-treated HepG2 cells (18.5 ± 1.5 µmol/mg protein).

### 3.2. Molecular Docking Studies and Binding to EGFR

Next, molecular docking studies were carried out to better understand the binding energies (Kcal/mol) and amino acid residue interactions of the most active isatin sulfonamide derivatives in the current study (**3a**, **4b**, and **4c**). The molecular docking results were compared to Dox and the co-crystalized ligand (Erlotinib) entering into the ATP binding site inside the active site of an EGFR (PDB: 1M17) retrieved from the protein data bank (https://www.rcsb.org/structure/1M17) (accessed on 5 February 2022) to explore the binding mode.

**Figure 5:**
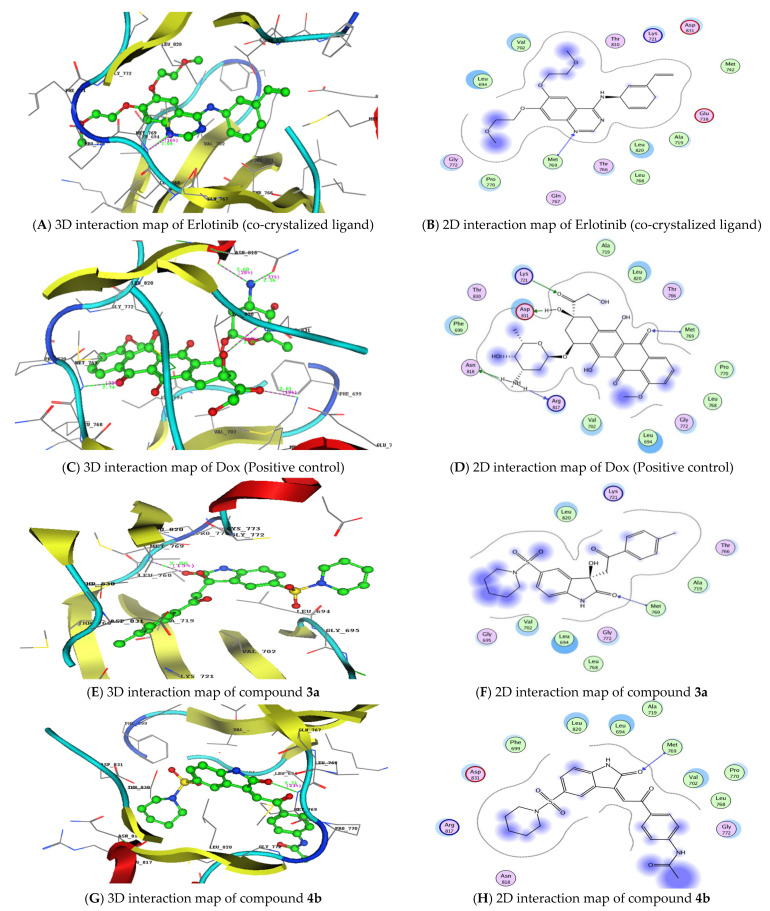

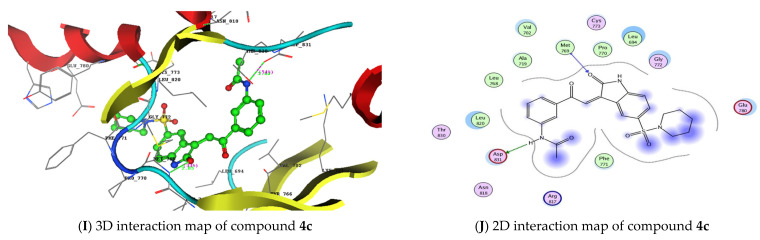
Molecular docking study: 3D and 2D interaction maps of the co-crystalized ligand (Erlotinib) (**A**,**B**), the positive control (doxorubicin) (**C**,**D**), and the isatin sulfonamide derivatives **3a**, **4b**, and **4c** (**E**–**J**) inside the EGFR (PDB:1M17) active site.

As shown in Table 3, **3a**, **4b**, and **4c**, when docked to the same binding site as Erlotinib (Table 5) revealed binding free energy with a minus score, showing quick fitting into the EGFR binding site, with free energies of −21.74, −19.21, and −20.80 kcal/mol, respectively. The results confirm the promiscuity of these three isatin sulfonamide derivatives to form good binding within the EGFR binding site [22,37]. Dox displayed lower binding energy of S = −22.82 Kcal/mol, through five hydrogen bonds, as did Erlotinib (S= −25.65 Kcal/mol). Compound **3a** displayed a hydrophobic interaction with piperidin-1-ylsulfonyl, phenyl derivatives of isatin’s bioactive core, as well as the carbonyl and tolyl groups (Figure 5). The hydrophobic interactions of both compounds **4b** and **4c** appear on the acetyl, piperidin-1-ylsulfonyl, and phenyl derivatives of the isatin scaffold. The synthesized isatin sulfonamide derivatives showed a hydrogen bond backbone acceptor between Met769 and a carbonyl of isatin with a low bond length of less than 3.04 Å, as did Erlotinib, that formed one hydrogen bond between the nitrogen of quinazoline and Met769, providing more evidence of good activity against EGFRs as promising new tyrosine kinase inhibitors.

## 4. Discussion

Molecular hybridization drug design based on combining different bioactive moieties into one compound is an effective strategy to produce promising efficient anticancer agents [17,46]. In the present study, molecular hybridization approach was used to synthesize ten new isatin sulfonamide hybrids. The anticancer antiproliferative activities of these derivatives, characterized using two HCC cell lines, HepG2 and Huh7, revealed that six compounds (**2a, 3a, 4a, 4b, 4c**, and **4d**) provided the most cytotoxic effects. The safety of these six compounds was investigated through in vitro and *in vivo* studies that showed that three derivatives (**3a**, **4b**, and **4c**) exhibited the least toxicity and higher safety margins.

To shed more light on the mechanistic pathways underlying the anticancer and antiproliferative activities of compounds **3a**, **4b**, and **4c**, we evaluated their effect on cell death tendency through studying cell cycle apoptosis. Our results showed that the compound “**3a**”, of all the tested compounds, has the highest cell cycle arresting potential during the cell cycle critical phases (M and pre-G1), rather than the other two new isatin hybrids (**4b** and **4c**) and the classically used cytotoxic positive control drug (Dox), in comparison to non-treated HepG2 cells (negative control). Cell cycle arrest percentage is now documented as a marker for new anti-cancer drug efficiency [47,48]. Our observations come in agreement with other studies reporting that some isatin derivatives arrested the cell cycle during the mitotic phase, in comparison to the positive cytotoxic control drugs cisplatin [49] and Dox [50].

One of the mechanisms by which anticancer agents could affect cell cycle machinery is through targeting various cyclin-dependent kinases (CDK_S_). Depending on the type of targeted CDK, cell cycle machinery could be arrested at different phases. Targeting cyclinA-CDK2 causes apoptosis induction at the S phase, while inappropriate activation of cyclinB-CDK1 targets the cell cycle at the G2-M boundary [48,51]. This could explain why isatin sulfonamide derivatives 3a and 4c arrested the cell cycle at the G2-M and pre-G1 phases, while 4b accumulated the cells at the G0-G1 and S phases. However, this needs more investigation by evaluating the effects of these derivatives on CDKs and the other enzymes regulating cell cycle machinery.

Regarding the mode of cell death analysis using Annexin V-FITC/PI double staining, it was found that compound **3a** had the highest induction of both total and late apoptosis in comparison to the **3b** and **4c** compounds, and it was even more potent than Dox. This suggests that compound **3a** would modulate cell death regulators and may play a key role in the decision of cell death having an apoptotic effect that would also be related to other cancer hallmarks [52,53].

This apoptotic efficacy raised by the **3a** compound was further supported by the significantly decreased expression of the survival anti-apoptotic protein (Bcl-2) compared to untreated HepG2. In addition, treating HepG2 with the **4b** and **4c** compounds caused a significant reduction in the Bcl-2 levels. Similarly, to our findings, different isatin derivatives could induce HepG2 cells death through targeting cell cycle proteins, apoptotic induction [50], and down-regulation of Bcl-2 with autophagy promotion [54].

Although **3a** had a much more potent apoptotic efficacy than the positive chemotherapeutic cytotoxic control Dox, the latter depressed the Bcl-2 level more than compound **3a**; this could possibly be due to the confined Dox effect against Bcl-2 solely [55].

Compounds **3a**, **4b**, and **4c** significantly decreased angiogenesis measured as EGFR protein, and the greatest reduction was observed when HepG2 cells were treated with **3a**. The potential of isatin sulfonamide derivatives to depress angiogenesis was also previously reported with apoptosis induction and arresting the cell cycle at the G2/M phases [56]. This inhibitory effect of the newly synthesized isatin sulfonamide derivatives (**3a**, **4b**, and **4c**) was confirmed by the molecular docking studies, as they displayed low free binding energy in comparison to the co-crystalized ligand (Erlotinib) and to the positive control (Dox) and inside the EGFR active site, meaning better potential anti-angiogenic drugs with tyrosine kinase inhibitory activity.

uPA is a highly restricted serine protease that converts the zymogen plasminogen to active plasmin, a broad-spectrum serine proteinase capable of degrading most protein components of the extracellular matrix, facilitating cancer invasion and metastasis (one of the cancer hallmarks) [57,58]. The three superior tested isatin sulfonamide-derivative compounds, **3a**, **4b**, and **4c**, showed possible anti-invasive activity measured as decreased uPA levels. Compound 3a showed the most significant efficacy that is comparable to the Dox effect. This observation is in accordance with a previously reported work, showing some isatin derivatives to decrease the cancer invasiveness in treated cancer cell lines via down-regulating urokinase [59].

The three currently investigated potential isatin sulfonamide derivatives, **3a** (the most superior), **4b**, and **4c**, significantly decreased the invasive potential of liver cancer cells through decreasing heparanase expression in the HepG2 cell lysate by a comparable degree to that shown in the Dox-treated HepG2 cells. Heparanase is an endo-β-D glucuronidase that cleaves an extracellular component, heparan sulfate. High expression levels of heparanase have been reported in several tumors [60,61] and associated with tumor growth, invasiveness, metastasis, and poor prognosis. In addition, there is crosstalk between heparanase and many other proteases such as matrix metalloproteinase-9 and uPA [62,63]. Therefore, downregulation of both uPA and heparanase by isatin derivatives (**3a**, **4b**, and **4c**) highlights their promising anticancer activities via targeting tumor invasion and metastasis.

It is worth mentioning here the role of heparanase in augmenting angiogenesis, another hallmark of cancer, through both its enzymatic and non-enzymatic activities. Release of heparan sulfate induces the angiogenesis via the upregulation of various growth factors and other proangiogenic factors [64,65]. Moreover, heparanase could promote angiogenesis non-enzymatically through activation of hypoxia-inducible factors, heat shock proteins, and proangiogenic factors [63,66]. Hence, the inhibitory effect of our newly synthesized isatin sulfonamide molecular hybrids on heparanase and EGFRs suggests a possible role in targeting angiogenesis.

Alongside this, we tried to explore the contribution of oxidative stress in the whole anticancer or apoptotic induction, through measuring both MDA and GSH levels. The tested isatin sulfonamide-derivative compounds did not affect the oxidative stress markers measured currently, and, together with their cytotoxic efficacy, did not rely on this stressful pathway.

In summary, the anticancer molecular or biochemical analysis revealed, mechanistically, that isatin sulfonamide derivatives’ cytotoxicity is possibly due to an effect on the apoptotic and the angiogenic machineries as well as targeting tumor invasiveness studied using the HepG2 cell line. The isatin sulfonamide derivatives showed an ability to depress the action of the survival mechanism of Bcl-2, inhibiting angiogenesis, and further hindering cancer invasion through inhibition of uPA and heparanase expression.

In future, more in vivo extrapolation for the compound **3a** will be conducted to introduce more insight into the anti-cancer autophagic/mitochondrial efficacy of the new molecularly hybridized isatin sulfonamide derivative, or, moreover, it will be engineered by nanotechnology for better drug targeting.

## 5. Conclusions

A schematic diagram is outlined in the graphical abstract, summarizing the present research’s findings on the newly synthesized isatin sulfonamide derivatives **3a, 4b**, and **4c**, which have promising anticancer activities on HCC cell lines. Moreover, the compound (**3a**) presents, hopefully, a promising new anticancer agent to suppress HepG2 cell growth in vitro through cell cycle arrest, apoptosis induction, and possible anti-angiogenic and anti-metastatic activities.

## Figures and Tables

**Table 1:** The in vitro cytotoxic activity of isatin sulfonamide derivatives on HepG2 and Huh7 cell lines (10 compounds) and in vitro safety assay towards normal human retina pigmented epithelial (RPE1) cell line (6 compounds) using MTT assay.

Cells	HepG2		Huh7		RPE1	
Compound no.	IC50 (µM)	% Death Rate at 100 p.p.m (μg/mL)	IC50 (µM)	% Death Rate at 100 p.p.m (μg/mL)	CC50 (µM)	% Cell Viability at 100 p.p.m (μg/mL)
2a	54.60 ± 2.00	81.50 *	40.00 ± 3.80	100 *	40.30 ± 1.61	4.40
2b	>100	10.20	>100	N.A	-	-
3a	16.80 ± 1.44	70.10 *	40.00 ± 2.20	100 *	>100	74 ^#^
3b	>100	1.20	>100	N.A	-	-
3c	>100	11.50	>100	N.A	-	-
3d	>100	19.50	>100	N.A	-	-
4a	12.00 ± 0.40	100 *	>100	N.A	21.90 ± 1.15	0
4b	44.70 ± 1.55	94.20 *	53.00 ± 3.00	76.00 *	>100	66.40 ^#^
4c	39.70 ± 1.90	95.60 *	35.00 ± 1.90	75.60 *	>100	80.70 ^#^
4d	13.30 ± 0.75	100 *	18.76 ± 0.8	0	11.90 ± 0.70	0
DMSO	>100	1	>100	5	>100	95
Doxorubicin	21.60 ± 0.81	100	11.60 ± 0.90	100	-	-

Results are expressed as the mean ± SEM of three separate experiments. IC50, half-maximal inhibitory concentration (used for HepG2 and Huh7 cell lines); CC50, half-maximal cytotoxic dose (used for healthy RPB1 cell line); N.A, no activity; ppm, parts per million (equivalent to μg/mL). * highly active compounds in case of HepG2 and Huh7 cells, and ^#^ highly safe compounds in case of RPE1.

**Table 2:** Effects of the acute oral toxicity study of isatin derivatives (**2a**, **3a**, **4a**, and **4b**, **4d**, and **4c**) on liver and kidney function tests of mice.

Tests		liver function				kidney function	
Parameters		S.ALT	S.AST	S.T.Bilirubin	S.D.Bilirubin	S.Creatinine	S.Urea
Groups	/Units	(U/L)	(U/L)	(mg/dL)	(mg/dL)	(mg/dL)	(mg/dL)
Control	Normal	28.92 ± 0.51	36.24 ± 0.94	0.69 ± 0.016	0.18 ± 0.005	0.67 ± 0.03	53.50 ± 1.01
	Negative	30.06 ± 0.72	34.40 ± 0.89	0.74 ± 0.016	0.19 ± 0.002	0.79 ± 0.03	52.38 ± 0.69
	Positive	52.97 ± 0.92 *^,#^	46.93 ± 0.30 *^,#^	1.45 ± 0.072 *,^#^	0.36 ± 0.026 *^,#^	1.57 ± 0.02 *^,#^	83.27 ± 0.95 *^,#^
Treated	**2a**	28.42 ± 0.27 ^^^	37.11 ± 0.35 ^^^	0.65 ± 0.018 ^^^	0.16 ± 0.003 ^^^	0.59 ± 0.04 ^^^	52.24 ± 0.65 ^^^
	**3a**	27.05 ± 0.81 ^^^	36.78 ± 0.33 ^^^	0.65 ± 0.026 ^^^	0.16 ± 0.002 ^^^	0.82 ± 0.03 ^^^	51.25 ± 0.36 ^^^
	**4a**	28.14 ± 0.56 ^^^	36.51 ± 0.15 ^^^	0.72 ± 0.019 ^^^	0.17 ± 0.004 ^^^	0.70 ± 0.02 ^^^	55.10 ± 2.96 ^^^
	**4b**	27.95 ± 0.34 ^^^	35.18 ± 0.47 ^^^	0.70 ± 0.013 ^^^	0.17 ± 0.001 ^^^	0.88 ± 0.06 ^^^	62.60 ± 3.64 ^^^
	**4c**	28.41 ± 0.28 ^^^	37.35 ± 1.47 ^^^	0.73 ± 0.014 ^^^	0.16 ± 0.007 ^^^	0.79 ± 0.04 ^^^	55.76 ± 5.64 ^^^
	**4d**	30.14 ± 0.93 ^^^	38.72 ± 1.37 ^#,^^	0.69 ± 0.016 ^^^	0.18 ± 0.004 ^^^	0.85 ± 0.03 ^^^	48.18 ± 0.53 ^^^

Data are expressed as mean ± SEM. Statistical analyses were carried out using ANOVA followed by the Tukey–Kramer post hoc test. Acute toxic doses of selected isatin derivatives (**2a**, **3a**, **4a**, and **4b**, **4d**, and **4c**, 250 mg/kg) were given to mice as a single oral dose, and the impact on their liver and kidney function tests was evaluated after 14 days of dose intake in comparison to normal healthy non-treated mice, DMSO-treated mice as negative control, and doxorubicin-treated mice as positive control (15 mg/kg, I.P, once). * *p* < 0.05 compared with the normal control; ^#^ *p* < 0.05 compared with the negative control group (DMSO-treated); ^ *p* < 0.05 compared with the positive control group (doxorubicin-treated).

**Table 3:** Cell cycle analysis using flow cytometry in HepG2 expressed as % of cells in each phase after treatment with isatin sulfonamide tested compounds, 3a, 4b, and 4c, and controls, and Annexin V/FITC apoptotic activity of selected isatin compounds is expressed as % of cell death induction.

Compound no.	%Cell Cycle Arrest/Phase				%Cell Death			
	%G0-G1	%S	%G2-M	%pre-G1	Total	Early	Late	Necrosis
					Apoptosis			
**3a**	31.57	23.58	44.85	46.29	46.29	3.56	29.58	13.15
**4b**	41.25	53.26	5.49	28.14	28.14	6.12	12.61	9.41
**4c**	36.44	25.94	37.62	32.02	32.02	2.88	18.08	11.06
Positive control	29.74	31.26	39	42.38	42.38	1.85	26.27	14.26
Negative control	55.29	37.61	7.1	1.47	1.47	0.36	0.12	0.99

Data are expressed as mean percentage of three separate experiments.

**Table 4:** Effects of treatment with the superior isatin sulfonamide derivatives (**3a**, **4b**, and **4c** compounds) on the levels of EGFR, uPA, BcL-2, heparanase, GSH, and MDA in cell lysate from HepG2 cells treated with the selected compounds at their IC50.

HepG2 Cell Line	Control		Treated		
Parameter/Groups	Positive	Negative	3a	4b	4c
EGFR (pmol/mg protein)	27.5 ± 1.7 ^a^*	306 ± 20	42 ± 2.3 ^a^*^,b^*	87.49 ± 3.4 ^a^*^,b^*	54 ± 2.4 ^a^*^,b^*
uPA (nmol/mg protein)	1244 ± 18 ^a^*	3149 ± 111	1258 ± 15 ^a^*	1916 ± 40 ^a^*^,b^*	1729 ± 38 ^a^*^,b^*
Bcl-2 (nmol/mg protein)	1.7 ± 0.10 ^a^*	7.8 ± 0.07	4.2 ± 0.10 ^a^*^,b^*	2.5 ± 0.04 ^a,b^*	3.6 ± 0.11 ^a^*^,b^*
Heparanase (pmol/mg protein)	902.1 ± 21 ^a^*	3097 ± 160	1262 ± 35 ^a^*^,b^*	1827 ± 30 ^a^*^, b^*	1449 ± 12.5 ^a^*^,b^*
GSH (µmol/mg protein)	0.8 ± 0.1 ^a^*	1.7 ± 0.2	1.6 ± 0.7	1.1 ± 0.7	2.1 ± 0.3 ^b^*
MDA level (µmol/mg protein)	18.5 ± 1.5 ^a^*	13.8 ± 1.0	2.0 ± 0.5 ^a^*^,b^*	3.7 ± 0.4 ^a^*^,b^*	14.5 ± 1.4

Data are expressed as mean ± SEM of three separate experiments. Results were computed by a one-way ANOVA test followed by Tukey–Kramer as a post-hoc test. * p values < 0.05 were considered significant. EGFR, epidermal growth factor receptor; uPA, urokinase plasminogen activator; Bcl-2, B-cell lymphoma; GSH, reduced glutathione; MDA, malondialdehyde. ^a^ difference from negative control (non-treated HepG2 cells); ^b^ difference from positive control (doxorubicin-treated HepG2 cells).

**Table 5:** Molecular docking study results of the isatin sulfonamide derivatives **3a**, **4b**, and **4c**, doxorubicin, and the co-crystalized ligand (Erlotinib), with binding energy and inter-acting groups with specific amino acids in residue inside the active site of the EGFR (PDB: 1M17).

**Compound no.**	**S (Kcal/mol)**	**Amino Acids Residues**	**Ligand Atoms**	**Distance (Å A)**	**Strength (%)**
**3a**	−21.74	Met769	C=O of isatin	2.86	15
**4b**	−19.21	Met769	C=O of isatin	2.71	23
**4c**	−20.80	Met769	C=O of isatin	2.85	33
		Asp831	NH of acetanilide	2.44	54
Doxorubicin	−22.82	Met769	C=O pf anthraquinone	2.74	32
		Lys721	C=O of ethenone	2.61	19
		Asp831	Hydroxy group	2.76	42
		Asn818	NH2 group	2.56	37
		Arg817	NH2 group	2.60	28
Erlotinib	−25.65	Met769	C=O of quinazoline	3.04	27

## Data Availability

Not applicable.

## Supplementary Materials

The following are available online at https://www.mdpi.com/article/10.3390/biomedicines10030722/s1. Synthesis and Identification of the Tested Isatin Sulfonamides Derivatives (10 Compounds).
