# Supplementary material for: Anticancer Effects with Molecular Docking Confirmation of Newly Synthesized Isatin Sulfonamide Molecular Hybrid Derivatives against Hepatic Cancer Cell Lines
Source: Biomedicines. 2022 Mar 20;10(3):722. doi: 10.3390/biomedicines10030722 (PMC8945686; doi:10.3390/biomedicines10030722)
Supplement: Supplementary file 1 [file biomedicines-10-00722-s001.zip › biomedicines-1606043-supplementary.pdf]

Supplementary File (S1)

Methodology

Synthesis and Identification of the Tested Isatin Sulfonamides Derivatives (10 Compounds)

All solvents and reagents were freshly distilled and purified according to the standard procedures. All melting points were recorded on digital Gallen Kamp MFB-595 instrument and corrected.

The IR spectra (KBr) (cm^{-1}) were measured on a Shimadzu 440 Spectrophotometer.

1H NMR spectra (δ, ppm) were obtained in deuterated dimethyl sulfoxide (DMSO-d6) and 13C NMR at 125MHz, spectra, obtained on a Varian Gemini 500 (500MHz) spectrometer, using TMS as an internal standard.

Chemical shifts were reported as δ ppm units.

The data were presented as follows: chemical shift, multiplicity (s=singlet, d=doublet, t=triplet, q=quartet, m= multiple, br=broad).

The IR spectra were recorded (KBr disk) on a PerkinElmer 1650 FT-IR instrument.

1H NMR (300 or 500 MHz) and 13 C NMR (75 or 125 MHz) spectra were recorded on a Varian spectrometer using DMSO as a solvent and TMS as an internal standard (app=apparent), coupling constant(s) in Hertz (Hz) and integration.

Mass spectra were recorded on Thermo Scientific ISQLT mass spectrometer at the Regional Center for Mycology and Biotechnology, Al-Azhar University. Elemental Analyses were carried out at the Micro Analytical Unit, NRC.

Synthesis of Arylidine (2a-b)

A solution of 5-(piperidin-1-ylsulfonyl) indoline-2, 3-dione (1) (0.01 mol) and active methylene (ethyl cyanoacetate and cyano acetamide) (0.01 mol) in absolute methanol (10 mL) containing catalytic triethylamine (3 drops), was stirred at room temperature for 0.5-2 h. The solid formed was collected and washed with methanol and left to dry, followed by recrystallized from proper solvent.

Synthesis of Ethyl (E)-2-cyano-2-(2-oxo-5-(piperidin-1-ylsulfonyl) indolin3-ylidene) acetate (2a) D10

Yield of 81.4 %, as red powder from ethanol, with melting point of > 300 °C; IR ν/cm^{-1}: 3305 (NH), 3086 (CH-Ar), 2985, 2935, 2854 (CH-aliph.), 2219 (CN), 1724, 1693 (C=O); 1H NMR (400 MHz, DMSO) δ/ppm 11.34 (s, 1H, NH; exchangeable with D2O), 9.45 (s, 1H, Ar-H), 7.66 (d, J = 5.6 Hz, 1H, Ar-H), 7.00 (d, J = 5.6 Hz, 1H, Ar-H), 3.88 (m, 2H, -CH2CH3), 2.84 (s, 4H, N-

2CH2-Pip.), 1.48 (s, 4H, 2CH2-Pip.), 1.28 (s, 2H, CH2-Pip.), 1.03 (s, 3H, -CH2CH3); 13C NMR (101 MHz, DMSO) δ 169.20, 165.57 (2C=O), 149.51, 148.07, 135.19, 133.71, 128.34, 121.61, 119.33, 114.21, 106.74, 54.28 (-CH2CH3), 46.85, 46.76, 24.95, 24.89, 23.12 (5CH2-Pip), 8.84 (-CH2CH3); Anal. Calcd for C18H19N3O5S (389.43) C, 55.52; H, 4.92; N, 10.79; Found: C, 55.71; H, 4.84; N, 10.64 %.

2-Cyano-2-(2-oxo-5-(piperidin-1-ylsulfonyl) indolin-3-ylidene) acetamide (2b) D11

Yield of 83.7 %, as red powder from methanol, melting point 280-282 °C; IR: ν/cm^{-1}: 3390, 3305, 3190 (NH+ NH2), 3081 (CH-Ar), 2985, 2935, 2854 (CH-aliph.), 2214 (CN), 1728, 1693 (C=O); 1H NMR (400 MHz, DMSO-d6) δ/ppm 11.47 (s, 1H, NH; exchangeable with D2O), 8.64 (d, 2H, NH, OH; exchangeable with D2O), 7.96 (s, 1H, Ar-H), 7.78 (d, J = 8.1 Hz, 1H, Ar-H), 7.13 (s, 1H, Ar-H), 2.87 (s, 4H, N-2CH2-Pip.), 1.56 (s, 4H, 2CH2-Pip.), 1.38 (s, 2H, CH2-Pip.); 13C NMR (101 MHz, DMSO-d6) δ 165.68, 161.98 (2C=O), 148.01 (C=C-N), 138.69, 133.96, 129.44, 125.23, 119.59, 114.89, 111.86, 111.69, 46.97, 25.12, 23.32 (5CH2-Pip.); Anal. Calcd for C16H16N4O4S (360.39) C, 53.32; H, 4.48; N, 15.55; Found: C, 53.54; H, 4.39; N, 15.61 %.

Synthesis of 3-hydroxy-3-(2-oxo-2-arylethyl)-5-(piperidin-1-ylsulfonyl) indolin-2-one (3a-d)

A solution of 5-(piperidin-1-ylsulfonyl)indoline-2,3-dione (1) (0.01 mol) and acetophenone derivatives (0.01 mol), was dissolved in methanol (10 mL) containing (0.5 mL) of diethylamine in a 100 mL conical flask and the mixture was stirred at room temp., until the solid product is formed (the progress of the reaction was monitored by TLC). The product formed was collected by filtration and crystallized from ethanol.

3-Hydroxy-3-(2-oxo-2-(p-tolyl)ethyl)-5-(piperidin-1-ylsulfonyl) indolin-2-one (3a) D1

Yield of 76.5%, as white powder from ethanol, melting point 238-240 °C; IR: ν/cm^{-1}: 3465 (OH), 3213 (NH-isatin), 3051 (CH-Ar), 2931, 2839 (CH-aliph.), 1712, 1681 (br C=O-isatin, acetyl); 1H NMR (400 MHz, DMSO-d6) δ/ppm 10.74 (s, 1H, NH, exchangeable with D2O), 7.75 (d, J = 7.6 Hz, 2H, Ar-H), 7.59 (s, 1H, Ar-H), 7.51 (d, J = 7.6 Hz, 1H, Ar-H), 7.25 (d, J = 7.2 Hz, 2H, Ar-H), 6.96 (d, J = 8.0 Hz, 1H, Ar-H), 6.29 (s, 1H, 1H, OH, exchangeable with D2O), 4.23 (d, J = 17.6 Hz, 1H), 3.48 (d, J = 17.2 Hz, 1H), 2.57 (s, 4H, N-2CH2-Pip,), 2.30 (s, 3H, CH3), 1.35 (s, 4H, 2CH2-Pip.,), 1.11 (s, CH2-Pip., 2H); 13C NMR (101 MHz, DMSO-d6) δ 196.73, 178.88 (2C=O), 147.73, 144.51, 134.01, 132.93, 130.10, 129.71, 128.61, 128.08, 123.40, 109.99, 73.26 (C-OH), 46.93 (CH2), 45.71, 24.89, 23.27 (5CH2-pipridinyl), 21.58 (CH3); Anal. Calcd for C22H24N2O5S (428.50): C, 61.67; H, 5.65; N, 6.54; Found: C, 61.45; H, 5.39; N, 6.62%.

3-(2-(3-Aminophenyl)-2-oxoethyl)-3-hydroxy-5-(piperidin-1-ylsulfonyl)indolin-2-one (3b) D2

Yield of 77.6 %, as red white powder from methanol, melting point 190-192 °C; IR: ν/cm^{-1}: 3411 (OH), 3410, 3356 (NH2, NH-isatin), 3055 (CH-Ar), 2935, 2889, 2835 (CH-aliph.), 1751, 1666 (br C=O-isatin, acetyl); 1H NMR (400 MHz, DMSO-d6) δ/ppm 10.73 (s, 1H, NH, exchangeable with D2O), 7.53 (m, 2H, Ar-H), 7.02 (s, 4H, Ar-H), 6.73 (s, 1H, Ar-H), 6.28 (s, 1H, OH, exchangeable with D2O), 5.29 (s, 2H, NH2, exchangeable with D2O-d6), 4.10 (s, 1H), 3.43 (s, 1H), 2.59 (s, 4H, N-2CH2-Pip.), 1.36 (s, 4H, 2CH2-Pip.), 1.16 (s, 2H, CH2-Pip.); 13C NMR (101 MHz, DMSO) δ 197.51, 178.89 (2C=O), 149.50 (C-NH2), 147.76, 137.32, 132.93, 130.09, 129.60, 128.11, 123.36, 119.27, 116.00, 112.93, 109.99, 73.28 (C-OH), 46.98 (CH2), 45.82, 24.98, 23.36 (5CH2-pipridinyl); Anal.Calcd for C21H23N3O5S (429.49): C, 58.73; H, 5.40; N, 9.78; Found: C, 58.58; H, 5.45; N, 9.89 %.

3-(2-(4-Aminophenyl)-2-oxoethyl)-3-hydroxy-5-(piperidin-1-ylsulfonyl) indolin-2-one (3c) D3

Yield of 73.6 % as yellowish white powder from ethanol, melting point 204-206 °C; IR: ν/cm^{-1}: 3460 (OH), 3352, 3228, 3132 (NH2, NH-isatin), 3078 (CH-Ar), 2943, 2924, 2843 (CH-aliph.), 1732, 1681 (br C=O-isatin, acetyl); 1H NMR (400 MHz, DMSO-d6) δ/ppm 10.72 (s, 1H, NH, exchangeable with D2O), 7.59 (m, 4H, Ar-H), 6.99 (s, 1H, Ar-H), 6.52 (s, 2H, Ar-H), 6.27 (s, 1H, OH, exchangeable with D2O), 6.15 (s, 2H, NH2, exchangeable with D2O), 4.09 (d, 1H), 3.44 (d, 1H), 2.62 (s, 4H, N-2CH2-Pip.), 1.42 (s, 4H, 2CH2-Pip.), 1.20 (s, 2H, CH2-Pip.); 13C NMR (101 MHz, DMSO-d6) δ 193.85, 179.05 (2C=O), 154.51 (C-NH2), 147.80, 133.15, 131.02, 129.99, 127.80, 124.33, 123.25, 112.88, 109.89, 73.50 (C-OH), 46.96 (CH2), 44.80, 25.10, 24.95, 23.32 (5CH2-pipridinyl); Anal. Calcd for C21H23N3O5S (429.49): C, 58.73; H, 5.40; N, 9.78; Found: C, 58.54; H, 5.41; N, 9.92 %.

3-hydroxy-3-(2-(4-hydroxyphenyl)-2-oxoethyl)-5-(piperidin-1-ylsulfonyl) indolin-2-one (3d) D4

Yield of 74.9 % as yellowish orange powder from ethanol, melting point 133-135 °C; IR: ν/cm^{-1}: 3398 (OH), 3302, 3197 (NH2, NH-isatin), 3068 (CH-Ar), 2970, 2917, 2858 (CH-aliph.), 1708, 1687 (br C=O-isatin, acetyl); 1H NMR (400 MHz, DMSO) δ/ppm 11.23, 10.76 (2s, 2H, NH + OH exchangeable by D2O), 8.06 (s, 1H, Ar-H), 7.85 (d, J = 8.4 Hz, 1H, Ar-H), 7.79 (d, J = 8.8 Hz, 1H, Ar-H), 7.60 (s, 1H, Ar-H), 7.01 (d, J = 8.0 Hz, 2H, Ar-H), 6.81 (d, J = 8.4 Hz, 1H, Ar-H), 6.31

(s, 1H, OH exchangeable by D2O), 4.20 (d, J = 16.7 Hz, 1H), 3.46 (d, J = 17.0 Hz, 1H), 2.82 (s, 4H, 2CH2-Pip.), 1.54 (s, 4H, 2CH2-Pip.), 1.38 (s, 2H, CH2-Pip.); Anal. Calcd for $C_{21}H_{22}N_2O_6S$ (430.48) C, 58.59; H, 5.15; N, 6.51; Found: C, 58.87; H, 5.02; N, 6.32%.

Synthesis of 5-(piperidin-1-ylsulfonyl)-3-(2-oxo-2-arylethylidene) indolin-2-one derivatives (4a-d):

Method 1: A mixture of 3-hydroxy-3-(2-oxo-2-arylethyl)-5-(piperidin-1-ylsulfonyl) indolin-2-one (3a-d) (0.001) mole, 0.5 mL of concentrated hydrochloric acid, and 15 mL of acetic acid was heated at 95°C on steam bath for 3 h. The mixture was allowed to stand at room temp., then the solid product was formed after poured on crushed ice, collected by filtration and crystallized from the proper solvent.

Method 2: A mixture of 5-(piperidin-1-ylsulfonyl) indoline-2, 3-dione (1) (0.01 mol) and acetophenone derivatives (0.01 mol) was dissolved in methanol (10 mL) containing (0.5 mL) of diethyl amine, stirred for 2h, added 10mL acetic acid with (1mL) hydrochloric acid, heated at 95°C on steam bath for 3 h, then the mixture was allowed to stand at room temp. The solid product formed after pouring on crushed ice was collected by filtration and crystallized from the proper solvent.

3-(2-Oxo-2-(p-tolyl)ethylidene)-5-(piperidin-1-ylsulfonyl)indolin-2-one (4a) D5

Yield of 72.9 % as yellow powder from ethanol, melting point 214-16 °C; IR: ν/cm^{-1}: 3217 (NH), 3051 (CH-Ar), 2931, 2839 (CH-aliph.), 1716, 1681 (C=O); 1H NMR (400 MHz, DMSO-d6) δ/ppm 11.32 (s, 1H, 2NH; exchangeable with D2O), 8.27 (s, 1H, -CH methine), 8.01 (d, J = 8.0 Hz, 2H, Ar-H), 7.82 (s, 1H, Ar-H), 7.72 (d, J = 8.4 Hz, 1H, Ar-H), 7.43 (d, J = 7.6 Hz, 2H, Ar-H), 7.11 (d, J = 8.4 Hz, 1H, Ar-H), 2.76 (s, 4H, 2CH2-Pip.), 2.42 (s, 3H, CH3), 1.52 (s, 4H, 2CH2-Pip.), 1.34 (s, 2H, CH2-Pip.); Anal. Calcd for $C_{22}H_{22}N_2O_4S$ (410.49) C, 64.37; H, 5.40; N, 6.82; Found: C, 64.54; H, 5.23; N, 6.98 %.

N-(4-(2-(2-Oxo-5-(piperidin-1-ylsulfonyl) indolin-3-ylidene) acetyl) phenyl) acetamide (4b) D6

Yield of 78.1 % as yellowish powder from ethanol, melting point 214-16 °C; IR: ν/cm^{-1}: 3344, 3109 (NH), 3092 (CH-Ar), 2939, 2854 (CH-aliph.), 1722, 1662 (C=O); 1H NMR (400 MHz, DMSO-d6) δ/ppm 11.53, 9.22 (2s, 2H, 2NH; exchangeable with D2O), 8.35 (d, J = 8.0 Hz, 1H, Ar-H), 8.25 (s, 1H, -CH methine), 8.07 (d, J = 8.4 Hz, 1H, Ar-H), 7.93 – 7.85 (m, 1H, Ar-H), 7.67 (s, 1H, Ar-H), 7.50 (d, J = 9.4 Hz, 1H, Ar-H), 7.32 (d, J = 8.3 Hz, 1H, Ar-H), 7.11 (d, J = 8.0 Hz,

1H, Ar-H), 2.87 (s, 4H, N-2CH2-Pip.), 2.37 (s, 3H, CH3), 1.54 (s, 4H, 2CH2-Pip.), 1.34 – 1.31 (m, 2H, CH2-Pip.); 13C NMR (101 MHz, DMSO-d6) δ 191.96, 183.47, 167.26 (3C=O), 159.94 (C=C), 154.14, 148.70, 140.62, 137.43, 135.08, 132.73, 131.63, 129.86, 128.80, 123.70, 120.68, 118.61, 113.21 (Ar-Cs), 47.06, 46.93, 25.09 (5 CH2-Pip.), 23.34 (CH3); Anal. Calcd for C23H23N3O5S (453.51) C, 60.91; H, 5.11; N, 9.27; Found: C, 60.75; H, 5.25; N, 9.14 %.

N-(3-(2-(2-Oxo-5-(piperidin-1-ylsulfonyl) indolin-3-ylidene) acetyl) phenyl) acetamide (4c) D7

Yield of 69.7 % as red powder from ethanol, melting point 160-162 °C; IR: ν/cm^{-1}: 3356, 3240 (NH-isatin), 3081 (CH-Ar), 2939, 2854 (CH-aliph.), 1728 (br C=O); 1H NMR (400 MHz, DMSO-d6) δ 11.17, 9.09 (2s, 2H, 2NH; exchangeable with D2O), 8.27 (d, J = 6.5 Hz, 1H, Ar-H), 8.01 (s, 1H, -CH methine), 7.97 – 7.89 (m, 1H, Ar-H), 7.96-7.92 (m, 1H, Ar-H), 7.76 (d, J = 10.2 Hz, 1H, Ar-H), 7.63 (s, 1H, Ar-H), 7.07-7.00 (m, 1H, Ar-H), 6.57 (d, J = 8.6 Hz, 1H, Ar-H), 2.85 (s, 2H, N-CH2-Pip.), 2.65 (s, 2H, N-CH2-Pip.), 2.05 (s, 3H, CH3), 1.50 (s, 4H, 2CH2-Pip.), 1.30 (s, 2H, CH2-Pip.); 13C NMR (101 MHz, DMSO-d6) δ 192.04, 183.45, 174.59 (3C=O), 159.96 (C=C), 155.65, 148.06, 139.17, 137.43, 133.32, 132.57, 132.18, 131.51, 130.93, 128.55, 123.70, 118.63, 113.49 (Ar-Cs), 47.06, 25.09 (5CH2-Pip.), 23.30 (CH3); Anal. Calcd for C23H23N3O5S (453.51) C, 60.91; H, 5.11; N, 9.27; Found: C, 60.85; H, 5.29; N, 9.18 %.

3-(2-(4-Hydroxyphenyl)-2-oxoethylidene)-5-(piperidin-1-ylsulfonyl) indolin-2-one (4d) D8

Yield of 71.3 % as yellow crystals from ethanol, melting point 214-16 °C; IR: ν/cm^{-1}: 3479 (OH), 3174, 3140 (NH), 3064 (CH-Ar), 2989, 2939, 2854 (CH-aliph.), 1716 (br C=O); 1H NMR (400 MHz, DMSO-d6) δ/ppm 11.29, 9.15 (2s, 2H, NH + OH; exchangeable with D2O), 8.57 (s, 1H, Ar-H), 8.28 (d, J = 8.8 Hz, 1H, Ar-H), 8.23 (d, J = 8.8 Hz, 1H, Ar-H), 8.08 (s, 1H, -CH methine), 7.99 (d, J = 8.7 Hz, 1H, Ar-H), 7.68 (d, J = 8.3 Hz, 1H, Ar-H), 7.09 (d, J = 8.3 Hz, 1H, Ar-H), 6.99 (d, J = 8.4 Hz, 1H, Ar-H), 2.99 (s, 2H, N-CH2-Pip.), 2.71 (s, 2H, N-CH2-Pip.), 1.57 (s, 4H, 2CH2-Pip.), 1.36 (s, 2H, CH2-Pip.); 13C NMR (101 MHz, DMSO-d6) δ 190.18, 167.45 (2C=O), 163.92 (C-OH), 150.09 (C=C), 148.39, 138.17, 134.09, 132.21, 129.80, 128.56, 127.58, 125.69, 122.82, 120.97, 116.43, 111.04 (Ar-Cs), 47.06, 46.93, 25.20, 25.07, 23.25 (5CH2-Pip.); Anal. Calcd for C21H20N2O5S (412.46) C, 61.15; H, 4.89; N, 6.79; Found: C, 61.51; H, 4.74; N, 6.88%.
